# The E-Nurture Project: A Hybrid Virtual Neonatal Follow Up Model for 2021

**DOI:** 10.3390/children8020139

**Published:** 2021-02-12

**Authors:** Paige Terrien Church, Rudaina Banihani, Jo Watson, Wan Ting Nancy Chen, Marilyn Ballantyne, Elizabeth Asztalos

**Affiliations:** 1 Department of Newborn and Developmental Paediatrics, Sunnybrook Health Sciences Centre, Toronto, ON M4N 3M5, Canada; Rudaina.banihani@sunnybrook.ca (R.B.); jo.watson@sunnybrook.ca (J.W.); nancy.chen@sunnybrook.ca (W.T.N.C.); Elizabeth.asztalos@sunnybrook.ca (E.A.); 2 Faculty of Nursing, University of Toronto, Toronto, ON M5T 1P8, Canada; Marilyn.ballantyne@hollandbloorview.ca

**Keywords:** neonatal follow-up, COVID-19, virtual clinic, social media, preterm infant

## Abstract

Neonatal follow-up has long focused on a model of surveillance and identification of short-term outcomes. This model has long become outdated, with evidence documenting the need for longer follow-up with known school-based challenges and significant gaps in knowledge by educators. This article reviews the history of neonatal follow-up and demonstrates a novel approach to neonatal follow-up, built largely with a hybrid virtual platform, which then became essential with the declaration of the pandemic in 2020.

## 1. Introduction

In 2000, *From Neurons to Neighborhoods* was published, calling into question the longstanding debate of nature versus nurture as independent influencers of childhood development, referring to the debate as “overly simplistic and scientifically obsolete” [1]. Instead, they called for these variables to be seen as dynamic and interactive. Twenty years later, amidst a pandemic, this review of neonatal follow-up care embraces this perspective of nature through nurture and describes how this principle can be integrated into a virtual neonatal follow-up clinic model focusing on neurodevelopmental programming.

Historically, neonatal follow-up care emerged with the intent to evaluate outcomes of those infants born preterm and admitted to the Neonatal Intensive Care Unit (NICU). The focus was on survival, followed by conditions associated with significant lifelong challenges, including cerebral palsy (CP), cognitive impairment, and neurosensory impairment, all of which can be identified in the first 3 years of life. As care changed over the years, the need to evaluate these outcomes remained relevant and timely with improvements in survival and long-term outcomes associated with improved neonatal care [2]. Over the last 15 years, however, neonatal care has stabilized, and the traditional outcomes of neonatal surveillance have also stabilized [3]. Of note, with the recent shift to provision of care for the incredibly preterm, or micropreterm gestation (i.e., those infants under 25 completed weeks), this surveillance remains relevant as the outcomes for this cohort are less certain and influenced by improved care practice [4].

Linked to the historical trend of improved outcomes is the improvement in brain injury/protection in NICU practice. Modifications to care with the introduction of gentle ventilation strategies [5], kangaroo care [6], greater attention to infection control [7], feeding practices [8], and head positioning [9] have resulted in less overt brain injury. What has, however, been recognized is that despite this prevention, there are fundamental differences in how the preterm brain develops in the altered environment of the NICU, with less cortical volume, less cerebellar development, and diminished white matter tracts. This has been referred to as dysmaturation [10].

In addition to the short range outcomes described above, there has been much work done to describe life for children born preterm in childhood, school years, and adulthood. This behavioral phenotype [11] has been described as one that is highly prevalent, affecting 50–70% of children born very preterm (under 32 weeks gestation) or very low birthweight (VLBW, under 1500 g at birth) [12]. It is characterized by a combination of challenges, including those with language comprehension and expression, social communication, learning, impulse control and busy behavior, organizational abilities, social immaturity, emotional lability, anxiety/internalizing conditions, and disorganized and immature motor coordination [11,12,13,14,15]. These challenges emerge after discharge from the traditional neonatal follow-up programs [11,12]. Unfortunately, they arise in the context of an educational sector ill-prepared to identify or intervene as educators have expressed a lack of knowledge about this behavioral phenotype [16,17].

With the absence of adequate knowledge and surveillance in the educational sector, a greater responsibility falls to the parents of the children born preterm to be aware of these potential challenges and have skills to advocate and teach their children strategies to navigate the world. This responsibility falls on the shoulders of individuals, however, who have a significantly greater likelihood of struggling with postpartum depression/anxiety [18] and posttraumatic stress [19], which can then impact the long-term outcome for the child [20].

The result appears to be a series of missed opportunities. Parents are struggling with their own mental health and trauma from the NICU [18], missing behavioral cues [21], or not being able to clearly interpret dysregulated cues coming from a child whose brain maturation [22] is altered by an atypical environment of the NICU followed by a transition to an educational system poorly equipped to accommodate them [11,17,18]. Combined with this is the absence of care from neonatal follow-up programs, with the majority ending at 2 or 3 years. [23,24] It is in this environment, in 2016, that we set out to redesign the delivery of neonatal follow-up care, drawing upon the literature from Neonatology but also heavily from that of Developmental Paediatrics.

## 2. Purpose of Neonatal Follow-Up

A critical first step from this review of the literature has been to revisit the goals of neonatal follow-up in the context of the stressors for the preterm child born less than 30 weeks and their family. The concept of surveillance remains an essential role of neonatal follow-up, particularly with the shift to care for peri-viable infants [4] as well as an important source of feedback to the NICU on outcomes. In addition, however, there was the added perspective of Developmental Paediatrics and its emphasis on what matters most to children and families, outcomes called the “F-words”: Fun, friends, family, fitness, function, and future [25]. When these “F-words” [25] become the focus, the goals of neonatal follow-up should then be to empower parents to know their children and advocate, to support extraordinary children regardless of their challenges, and to espouse hope [26]. With this as the driving force, the following changes were made.

## 3. Proposal for Neonatal Follow-Up 2021

The schedule of visits, rather than assigned arbitrarily, focuses on specific touchpoints (Table 1) [27]. Touchpoints describe moments of child development that may cause disruption in the family. They are positive moments for identification and intervention and education. Once we identified touchpoints, the tools to assess, and goals for education and intervention could then be articulated.

Three principals were foundational to this redesign and shift in the structure. First, surveillance for the traditional outcomes of prematurity remains necessary to identify outcomes associated with disability: cerebral palsy, cognitive and neurosensory impairment. The goal of identification, while important for NICU quality assurance and counseling, however, is in light of the evidence for the functional benefits of early intervention and identification [28,29,30]. It then shifts the focus to optimizing the “F words” [25]. These conditions often present early, and assessments with the best evidence guide the identification process [28] when possible. In addition, the commitment to collaboration nationally and internationally is maintained with standardized data collection. The second principle should be to fill gaps in the system of care, rather than duplicate services. Collaboration with the community and school resources, as well as the parents, is essential to avoid duplication and maintain consistency in messaging and care. Lastly, the third principle reflects the challenge of the behavioral phenotype of prematurity, manifesting inconsistently and in potentially multiple domains. This pattern has made extended follow-up challenging as assessing all domains is cost-prohibitive, time-intensive, and exhausting for the child. [31] Rather, we propose that the extended, less traditional neonatal follow-up visits be driven by touchpoints combined with identified gaps in the systems of care/education. These include the transition to school, with the touchpoints of peer relationships and social development, which have been ascribed as a key school readiness skill [32]. The other identified high yield touchpoint is to focus on academic learning and early identification of barriers to learning in the early school years (Table 1).

Operationalizing these principles included the selection of tools. The factors driving tool selection (see Table 1) started with the identified touchpoint, and objectives such as these offered the primary focus for the visit. In addition, the factor of staffing in the clinic and training in keeping with various grades of skill required for specific developmental assessments. Lastly, the variable of cost was considered with preference given for free tests as possible.

## 4. A Data Informed Push toward Virtual

Critical to this process is engagement with parents as major stakeholders. In order to do this, parents enrolled in the clinic as well as those with infants in the NICU, received a survey inquiring about their perceptions of the ideal format for support and resources that would be useful in the community. The responses called for access to educational content with the ideal forum identified as web-based, professionally run with a focus on local resources. This approach is supported in the literature [33]. Onsite workshops for parents were less desired as they present a barrier due to limited time and extra costs associated with parking and babysitting.

The educational web-based supplement to the clinic visits needed to have a dynamic component in order to provide time-sensitive information as well as respond to questions. Social media is an ideal vehicle for this goal, focusing on Facebook ™ given the high volume of users amongst parents with the commonly stated goal of seeking support and education [34]. With consultation with hospital communication and privacy, a local Facebook site has been piloted, and a curriculum designed with monthly topics spanning the age ranges of clinic participants. We developed content based on evidence and written by team members. Participation has been voluntary and parent initiated, with the administration of the site run by team members from the clinic. Once parents have answered security questions to join, clarifying that they have a child enrolled in the clinic, they can choose to be anonymous or to remain identified. Daily posts have resulted in an increase in membership of 149% from 1 January 2020 to 1 December 2020. A review of member activity reveals that between 58 and 78% are active members, depending on topic [35]. Preliminary satisfactory data from this group demonstrated that membership was predominantly from those parents of preterm children, mean gestational age 26 weeks. Of those that offered data on gender, the site was utilized more by females 97% over males 3%. Parents rated the site as useful–extremely useful; no parent rated the site as not useful.

In addition, specific areas of focus for programming identified by the clinical team included gaps in community resources with social communication [36] and an increased prevalence of autism spectrum disorder (ASD) [37] identified and linked to potential future social challenges [15]. With feedback from parents, the goal was to explore this programming to be offered online in order to be widely accessible. The Hanen Centre, which is an international resource for early language and communication, has developed an online version of their program for social communication [38], called More Than Words. The online component utilizes a Hanen certified instructor to coach parents over an 8 week time period on strategies to facilitate language and social communication skills. Implementation of this program is in its pilot phase in our clinic, with the first group running currently.

## 5. COVID-19

In March 2020, a pandemic was declared by the World Health Organization (WHO) caused by severe acute respiratory syndrome coronavirus 2, or COVID-19 [39]. As a result, all clinical care that was deemed nonessential was canceled. This included developmental surveillance for neonatal follow-up across the world. Many clinics have adapted with differing degrees of virtual care, but with the above changes in place, the shift for this clinic was nimble and immediate. No patients have been canceled due to COVID-19, rather appointments transitioned to virtual platforms. Enrollment per year in the program is 220 children per year. In comparison to monthly attendance data from 2019, the attendance rate has increased from 75% to 85–90% consistently, and the overall volume of visits has also increased by 25%. Visits have been accomplished in an interprofessional manner, with medical and therapy collaboration, using videoconferencing technology. Consent forms satisfactory to the medical, nursing, occupational therapy, speech therapy, and physiotherapy regulatory colleges were established and are provided prior to video conferencing. Transition to home has been improved with the ability for the follow-up team to now do a virtual visit, seeing into the family’s home, observing feeding, and answering questions. Infant growth is monitored with parents providing weights and feeding histories combined with virtual feeding assessments. Infant surveillance has remained possible with the video-based tool, Assessment of General Movements [28]. The transition to school and school-aged visits have been conducted with minimal disruption as the data collected can be accomplished virtually (see Table 1).

With the platform of a website and Facebook ^TM^ established, internet-based resources have been further augmented. Videos for motor-based coaching are available and are shared via email and posted online for parents. A weekly program of a virtual drop-in session with clinic members and parents of children enrolled in the clinic has also been established as a social support and information session. An ‘ask the doctor’ session is held on Instagram ^TM^ monthly with parents’ questions mainly on feeding, COVID-19, and behavior challenges being answered. The responses were then videotaped or typed and available to all members. Lastly, collaboration with community resources has been established virtually, with community providers participating in video visits, thereby ensuring consistency of messaging and offering further coaching virtually.

Limitations have been identified. The administration of the Bayley Scales of Infant Development has not been possible to be implemented virtually, despite the BSID IV having the added option of ‘report by parent’ [40]. From the review, the report by parent component of the BSID IV appears to provide approximately 15–20% of the needed data. While the option of video conferencing provides tremendous insight into the family, home, and feeding, it cannot replace the benefit of a physical examination of a child.

## 6. Conclusions

### Future of Neonatal Follow-Up

While the pandemic will end, and care will return to ‘normal,’ the lessons learned from decades of experience in neonatal follow-up, combined with those most recently from COVID-19, provide the opportunity to improve care and services. The redesign of neonatal follow-up is essential, now more than ever, and the redesign recommendations reviewed are feasible, efficient, and evidence-based. The evidence around outcomes [3,11,12,13,14,15,18,19,20,21,22], benefits of identification [28], and intervention [29,30], and gaps in system [16,17] are all well recognized and documented. The cost of raising a child with a developmental disability ranges from $250,000 to $2.4 million USD [41], and the preterm represents 8% of all live births [42] with a 50–70% likelihood of challenges in school [11,12] and 10–15% having significant challenges [3]. This population, therefore, does contribute to a significant societal cost. The nature of dysmaturation, however, underscores that it is not overt injury [10] but rather alterations to development, the nature of which offer potential for plasticity with nurture [1].

In order to improve neonatal follow-up care, what is clearly needed is an appreciation for the gaps in the systems of care [11,16,17,23,24] as well as an appreciation for how parents prefer to access information and services [33]. In addition, there is the need for a shift in focus from ‘surveillance to identify’ to ‘surveillance to optimize’ [25] with the intervention [28,29,30]. In doing so, the NICU and neonatal follow-up community will continue to understand NICU practice and impact on outcomes through the data collected, but we will also see a shift in perspective from one of looking for what is ‘wrong’ and celebrating all that is and can be ‘right’ [25,43]. We will also see a shift from identifying what systems are not working [16,17,23,24] to a system of parents empowered, educated, and capable of supporting their own children.

## Figures and Tables

**Table 1:** Touchpoint and visit schedule.

Age at Visit	Touchpoint	Objective	Assessment	Critical Team Members
1 week	Making a place in the family	1. Check feeding status 2. Check growth 3. Review parental mental health 4. Start parent education about their baby, what is new, different, unique, and how to manage	Virtual feeding assessment Weight Edinburgh Postnatal Depression Scale	Medical/nursing Therapy
4–6 weeks CA	Mental health of parents	1. Follow-up mental health 2. Initiate surveillance for cerebral palsy 3. Parent education	EPDS Generalized Anxiety Disorder Scale Assessment of General Movement	Medical/nursing Therapy
4 months CA	Roles established in family and rituals needed	1. Family wellbeing 2.Early evaluation of motor skills and rituals of exercises provided 3. Feeding/sleeping rituals to be established 4. Parent education	Surveillance of family AGM Hammersmith Infant Neurological Exam Movement Assessment of Infants	Medical/nursing Therapy
8 months CA	Emerging independence	1. Motor skills and safety 2. Feeding transitioning to eating 3. Emerging regulation 4. Parent education	HINE MAI Survey of Well Being in Youth Children	Medical/nursing Therapy
12 months CA	Stepping up and out	1. Motor and safety 2. Emerging social skills 3. Behavioral management introduction 4. Community growing with child—no longer only family influences	SWYC	Medical/nursing Therapy
18 months	Independence and limits	1. Behavioral management in the face of greater independence 2. Social communication skills screening and assessment for Autism Spectrum Disorder 3. Data collection for networks	SWYC Bayley Scales of Infant Development-III/IV ±Autism Diagnostic Observation Schedule-2nd Edition	Medical/nursing Therapy
3 years	Preschooler sense of self	1. Behavioral management in the face of school transition 2. Social communication and self-regulation 3. Developmental assessment for extreme preterm gestations	SWYC BSID III/IV	Medical/nursing Therapy
4–5 years	Peer relationships	1. Transition to school and success with social skills/peer relationships 2. Ability to follow structure and rules 3. Parent education	SWYC Canadian Paediatric Society, Preschool/Kindergarten Teacher questionnaire Canadian Paediatric Society, Preschool/Kindergarten Parent questionnaire ±Further development/motor assessment for suspected concerns interfering with school function	Medical Therapy
7–8 years	Learning skills necessary Peer relationships	1. Screening of learning skills and achievement 2. Peer relationships 3. Parent education	SWYC Canadian Paediatric Society, School age Teacher questionnaire Canadian Paediatric Society, School age Parent questionnaire Vanderbilt ADHD Diagnostic Rating Scale (VADRS) Kaufman Brief Intelligence Test Kaufman Test of Educational Achievement ±psychoeducational assessment in community if concerns identified	Medical Therapy

CA = corrected age; EPDS = Edinburgh Postnatal Depression Scale; GAD-7 = Generalized Anxiety Disorder Scale 7; AGM = Assessment of General Movements; HINE = Hammersmith Infant Neurological Exam; MAI = Movement Assessment of Infants; SWYC = Survey of Well Being in Youth Children; BSID III/IV = Bayley Scales of Infant Development III or IV; ADOS-2 = ± Autism Diagnostic Observation Schedule-2nd Edition; CPS = Canadian Paediatric Society; VADRS = Vanderbilt ADHD Diagnostic Rating Scale; KBIT = Kaufman Brief Intelligence Test; KTEA = Kaufman Test of Educational Achievement.
